# A case of pulmonary ALK-positive histiocytosis combined with Birt-Hogg-Dubé syndrome carrying an *EML4::ALK* gene fusion: a case report and literature review

**DOI:** 10.3389/fimmu.2024.1501217

**Published:** 2025-01-10

**Authors:** Shuzhe Yang, Zhongyuan Bai, Qi Zhao, Yajing Wang, Yanfeng Xi

**Affiliations:** ^1^ Second Clinical Medical School, Shanxi Medical University, Taiyuan, China; ^2^ First Clinical Medical School, Shanxi Medical University, Taiyuan, China; ^3^ Department of Pathology, Shanxi Province Cancer Hospital/Shanxi Hospital Affiliated to Cancer Hosipital, Chinese Academy of Medical Sciences/Cancer Hospital Affiliated to Shanxi Medical University, Taiyuan, China

**Keywords:** ALK-positive histiocytosis, Birt-Hogg-Dubé syndrome (BHD), EML4- ALK, FLCN gene mutation, case report

## Abstract

In this article, we report the first case of a 61-year-old woman who was diagnosed with both nodules and cystic lesions in her lungs. The lung nodules were diagnosed as ALK-positive histiocytosis (APH) carrying an *EML4::ALK* gene fusion, which microscopically displayed a mixed morphology of foamy cells, spindle cells, and Touton’s giant cells. Immunohistochemistry showed expression of CD163, CD68, and ALK, while fluorescence *in situ* hybridization (FISH) with second-generation sequencing (NGS) showed the ALK gene fusion with the FLCN gene variant. The patient also had bilateral multiple cystic lesions in the lungs, which were morphologically consistent with pulmonary bullae. The FLCN gene variant, in combination with the results of NGS, led to the diagnosis of Birt-Hogg-Dubé syndrome (BHD). APH and BHD are very rare, and it is easy to misdiagnose or miss the diagnosis altogether if one is not familiar with the associated histology and immunohistochemistry. It is essential for pathologists to recognize the presence of these two diseases and understand the associated histomorphologic, immunohistochemical, and cytogenetic features to enable an accurate diagnosis and differential diagnosis.

## Introduction

ALK-positive histiocytosis (APH) is an unusual type of histiocytic neoplasm that can affect organs such as the liver and nervous system, as well as the spleen and bone marrow. While APH is often associated with involvement in various organs, the presentation of APH as an isolated lung nodule is extremely rare. In the available reported literature on the subject, there is great variability in the age, site of onset, and pathohistologic features of patients who develop APH. The treatments offered to different patients and their prognosis are also very different. Therefore, it is crucial to study the prognosis and treatment of patients with APH occurring at rare sites. Multisystemic patients usually receive systemic therapy with ALK inhibitors with favorable results. Systemic therapy based on surgical resection is more recommended for monosystemic patients, but there are still patients who have experienced disease progression. On the other hand, BHD is a rare autosomal dominant disorder caused by germline mutations in the follicular proteins encoding tumor suppressor gene FLCN. Typically, BHD presents as multiple pulmonary cysts with or without recurring spontaneous pneumothorax when it occurs in the lungs. This article is the first to report the combined existence of APH and BHD, and pathologists need to pay close attention to them. In conclusion, this case demonstrates the importance of recognizing the existence of two rare diseases, APH and BHD, and understanding their associated features. Notably, *EML4::ALK* fusions are common due to inflammatory myofibroblastic tumor (IMT) occurring in the lung. Care needs to be taken to differentiate it from IMT when an inflammatory background with spindle cells is seen on APH microscopy. Immunohistochemistry was positive for myogenic markers such as SMA and Desmin in IMT, whereas APH did not express these markers. With improved awareness and knowledge, pathologists can more accurately diagnose and treat these rare diseases.

## Case description

A non-smoking 61-year-old woman presented in August 2023 with fever, dry cough, chest tightness, shortness of breath, and pain. The fever resolved after three days of self-medication but recurred. On September 22, 2023, a chest CT scan revealed multiple bullae in both lungs, nodules in the right lower lobe. Because dendritic cell and histiocyte tumors rarely occur in the lungs, the imaging specialist diagnosed this patient with IMT, but it was actually clinically difficult to rule out a diagnosis of Langerhans’ cell histiocytosis. She underwent a right lower lobe wedge resection on October 19, 2023 (**Table 1**).

**Table 1:** Timeline-Historical and current information from this episode of care organized as a timeline.

Time	August 2023	September 22, 2023	October 19, 2023
current information	fever, dry cough, chest tightness, shortness of breath, and pain	a chest CT scan revealed multiple pulmonary bullae in both lungs, nodules in the right lower lobe	She underwent a right lower lobe wedge resection

The resected nodule, 2.5 cm in diameter, had a soft, greyish-white texture and was near the pleura. Microscopic analysis showed irregular polygonal foamy histiocytes, spindle cells, multinucleated and Touton giant cells, thin-walled blood vessels, and some lymphocytes. Immunohistochemistry showed that these foam cells along with spindle cells expressed positive markers (CD68, CD163, CD4, ALK-D5F3, vimentin, CD10, caldesmon, CD99, calretinin nuclei, cyclin D1) and negative markers (S100, SMA, Desmin, CD1a, Langerin, B-raf,β-catenin, AE1/AE3, CK5, CK7, P40, TTF1, Napsin-A, Syn, CgA, Melan-A, HMB45, Hepatocyte, CD30, Inhibin, PAX-8). A commercially available LSI break-apart probe set for the ALK gene (Vysis, Abbott Molecular; 3’-ALK Spectrum Orange, 5’-ALK Spectrum Green) was used for FISH analysis. ALK gene fusion was demonstrated by counting at least 100 cells and observing a positive signal (segregating and/or segregating 3’signals) in 15% of the tumor cells. FISH confirmed ALK gene fusion, diagnosing ALK-positive histiocytosis (APH). Additionally, cystic lesions in the right lung exhibited enlarged alveolar foramina, fractured septa, and fused dilated bullae. It is very easy to underdiagnose BHD because the patient does not present with the typical BHD syndrome of fibrofolliculomas and other clinical symptoms. Second-generation sequencing identified an *EML4::ALK* gene fusion with a FLCN gene variant, confirming APH combined with BHD. We completed the NGS assay based on the IIIumina platform and hybridization capture method. Of these, the FLCN variant allele frequency(VAF) was 69.29%, while the VAF for ALK was 22.76%.

**Figure 1:**
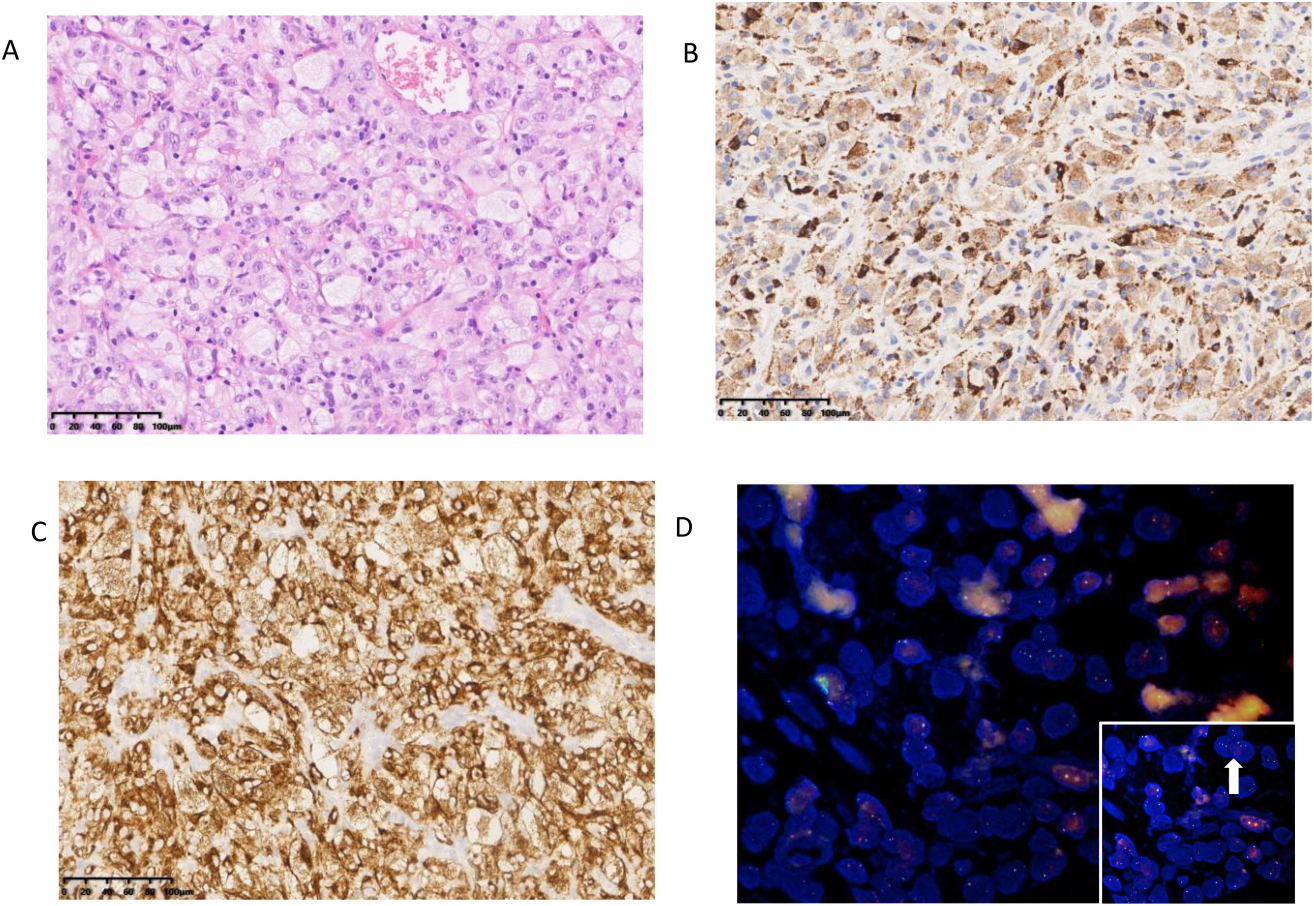
Pathological features of case **(A)** Lung nodules present as large pleomorphic histiocytes in the form of foam cells and spindle cells. **(B)** The tumor cells were positive for CD68. **(C)** The tumor cells were positive for ALK. **(D)** ALK-FISH (a break-apart probe) was positive.

During follow-up, no further treatment was given, and there was no evidence of recurrence or metastasis at the 8-month follow-up (**Figure 1**).

## Discussion

APH is a rare subtype of histiocytic tumor, characterized by the accumulation of macrophages, dendritic cells, or monocytes (1). APH exhibits a rich and diverse range of histological morphologies and manifests slightly differently across various organs. Most cases display the classic xanthogranuloma features, with microscopy revealing foamy macrophages and Touton giant cells, and sometimes epithelial-like cells. The foamy cells are eosinophilic with oval-shaped nuclei, which may show slight nuclear membrane folding or indentation; mitotic figures are rare. Some spindle cells are also seen, which have eosinophilic cytoplasm, indistinct borders, and ovoid or elongated nuclei. The proportion of these two types of cells varies considerably in different sites of disease, making it essential to have clear diagnostic criteria. In the latest WHO classification, APH is categorized as a histiocytic neoplasm of the lymphohematopoietic system (2). Diagnosis requires histiocytic aggregates, sheet-like tissue infiltration, and no dysplasia. Confirmation involves two or more histiocytic markers (e.g., CD163, CD68, CD14, CD4, lysozyme) and positive ALK staining. Key diagnostic features include irregularly folded nuclei and ectopic ALK gene expression.

Less than 100 cases have been reported to date. The condition is more common in females than in males and can occur in individuals ranging from infants to adults. The clinical manifestations of APH are closely related to the age of the patient. In infants, APH primarily presents as a systemic disease affecting the liver and hematologic system, characterized by hepatomegaly, anemia, and thrombocytopenia. Such patients received supportive therapy including blood transfusion and we observed spontaneous regression of the disease. Another patient received second-line systemic therapy with dexamethasone, anabolic acid and azathioprine and achieved complete remission. The remaining patients showed varying degrees of progression after receiving treatment regimens such as chemotherapy, but the effectiveness of the various treatments is still in the research phase because too few patients participated in the study. In children and adults, APH can involve the hematologic system, nervous system, as well as bones, lungs, liver, skin, and lymph nodes, often presenting as a disseminated disease affecting one or multiple organs (3). For patients with multisystem involvement, first-line treatment is conventional systemic therapy alone, which may be combined with an ALK inhibitor. For patients with unisystemic involvement, surgical resection is necessary, along with conventional systemic therapy. Pulmonary APH lacks typical clinical symptoms and may be detected through imaging as solitary pulmonary nodules (4). It can also present as multiple pulmonary nodules accompanied by involvement of the nervous system (5). Patients with nervous system involvement exhibit a variety of neurological symptoms, including seizures, ataxia, headaches, and vomiting. When APH only involves the skin, the tumor characteristics are very similar to those of cutaneous JXG, and the difference between the two lies mainly in the presence or absence of an ALK translocation. A recent study showed that these cases are basically xanthogranulomas with ALK fusion (6).

In this case, the spindle cells tested positive for Caldesmon and negative for SMA and Desmin, possibly due to Caldesmon’s higher specificity (7). We believe that this situation may be related to the tendency of the spindle cells in the tumor tissue of this patient to have smooth muscle differentiation. Existing literature reports that the smooth muscle differentiation of gastrointestinal stromal tumor (GIST) cells is associated with *EML4::ALK (* 8). We hypothesized that ALK rearrangements may contribute to an immunophenotype of APH tumor cells similar to that of spindle cells expressing myogenic markers within IMT. This could explain the histological and immunophenotypic similarities to IMT, such as spindle cell morphology and Caldesmon expression. Caldesmon expression was seen in IMT, thus IMT needed to be ruled out before diagnosing APH in this patient. In this case, the tumor cells were negative for SMA and Desmin in IHC, which led to the final diagnosis of the case as APH. The treatment of IMT is consistent with that of APH, and surgery is considered the mainstay of treatment. If complete resection is not possible due to anatomical location or comorbidities, medical therapy combined with radiation therapy may be considered (9). CD68 and CD163 expression varied across tumor sections, with some showing strong positivity and others resembling atypical ALK-rearranged histiocyte-rich tumors (3). The exact cause of this variation is unclear, and it remains uncertain whether atypical ALK-rearranged histiocyte-rich tumors and APH are identical. Pathologists should examine multiple sections and use immunohistochemical staining to confirm an APH diagnosis, especially if CD68 and CD163 staining does not align with diagnostic criteria.

### Molecular genetical features

APH is primarily distinguished by ALK rearrangement, which may activate downstream pathways and drive cells into the cell cycle. In ALK fusions like KIF5B-ALK, the fusion of an amino-terminal chaperone with the ALK tyrosine kinase domain activates the RAS/RAF-MEK-ERK (MAPK) and PI3K/AKT/mTOR signaling pathways. This activation leads to ERK phosphorylation, promoting transcription of genes such as CCND1 (3). Due to the small number of patients with APH, the gene fusion of *EML4::ALK* has been detected in no more than ten patients in the available studies. Among the available cases, *EML4::ALK* fusions have been detected only in patients with APH in the involved lungs. The age of these patients was not clearly characterized and could occur in children and adolescents as well as in middle-aged and elderly people. And no significant differences in morphology were seen between APH that developed *EML4::ALK* fusion and those that developed fusions of other partner genes. The role of EML 4-ALK fusion in the pathogenesis, treatment, and prognosis of ALK-positive histiocytosis, especially in patients with isolated lung involvement, still needs to be further explored (10–14).

The patient also underwent FLCN gene mutation testing. Confirmation of a BHD diagnosis relies on a combination of clinical features and/or FLCN germline mutation identification (15). The presence of these mutations, combined with multiple pulmonary cysts, led to a diagnosis of BHD, a rare autosomal dominant disorder caused by germline mutations in the FLCN gene, which encodes follicular proteins. Diagnosis is primarily clinical due to the lack of specific microscopic features. BHD typically presents with pulmonary cysts, spontaneous pneumothorax, fibrofolliculomas, and various renal tumors (16).

Upon communication with the patient, it was learned that the patient’s father and sister had both suffered multiple episodes of pneumothorax. BHD manifests itself as a rare autosomal dominant disorder, and its clinical manifestations usually include recurrent spontaneous pneumothorax. Since the patient’s father has passed away we performed NGS on blood samples from the patient and her sister. The test results showed that both of them had a VAF of 50% for the FLCN variant. This result confirmed the presence of FLCN gene germline mutation in the patient. For such cases, second-generation sequencing is recommended to identify FLCN variants. Regular renal monitoring is also advised due to BHD’s association with renal tumors. Given the patient’s FLCN mutations and medical history, we hypothesize that APH may have developed secondary to cystic lesions from BHD. This is the first reported instance of both conditions in one patient, necessitating further investigation into their potential interaction.

### Differential diagnosis

APH must be distinguished from several other conditions by combining clinical history with morphological and immunohistochemical features. APH needs to be differentiated from juvenile xanthogr-anuloma (JXG), Rosai-Dorfman disease (RDD), Erdheim-Chester disease (ECD), IMT, reticulohistiocytoses(RH) and Histiocytic Sarcoma (**Table 2**).

**Table 2:** Differential diagnosis of APH.

	Clinical features	Histological features	IHC and molecular features
JXG	JXG is usually confined to the skin in children. It rarely involves extracutaneous tissues or systemic organs, and when it does it can lead to high morbidity and mortality.	JXG is classified into early (EJXG), classic (CJXG), and late (TJXG) subtypes. EJXG features sheet-like histiocyte infiltration with minimal lipid vacuoles. CJXG shows more vacuolated histiocytes and occasional multinucleated giant cells. TJXG is characterized by predominance of spindle cells and foamy giant cells.	Of note, a recent study performed molecular testing on a subset of pediatric patients diagnosed with JXG. The results showed that five out of eight patients (62.5%) with pulmonary involvement carried the ALK translocation. In contrast, the above molecular alterations were not detected in patients with skin-only occurrence. This conclusion suggests that pathologists should not distinguish JXG from APH only by the presence or absence of ALK translocation, but also by the age of the patient, the location of the disease. JXG’s morphology and ALK negativity aid in distinguishing it from APH (17).
RDD	This rare non-Langerhans histiocytosis mainly affects children and young adults. RDD, also known as sinus Histiocytosis with massive Lymphadenopathy (SHML), is a rare benign entity of unknown etiology. RDD rarely involve extranodal sites independent of the lymph node status. RDD occurring in the lungs is extremely rare and, at present, there are only very few reports of this disease in the literature (18).	Histologically, it features enlarged nuclei, prominent nucleoli, and abundant cytoplasm with phagocytosis of inflammatory cells.	Tumor cells can be positive for CD68 in IHC. RDD is distinguished from APH by tumor cells expressing S100 and OCT2 and excluding ALK (19).
ECD	Notable for long bone involvement and distinctive kidney appearances on CT.	ECD often presents with foam cell infiltrates and surrounding fibrosis or granulomas. The presence of Touton giant cells is common but not universal.	Tumor cells expressed CD68, CD163, Factor VIIIa, and CD14 and did not express S100, CD1a, and Langerin. BRAF V600E mutations are common in patients with ECD (20).
IMT	While most commonly arising in the lung and deep intra-abdominal soft tissues, IMT has been reported at a wide variety of body sites. Lung IMT may be associated with cough, dyspnea, and chest discomfort, and soft tissue IMT typically presents as an asymptomatic abdominal mass.	IMT usually appears as well-defined, lobulated masses with a grayish-white hue, prominent fibrosis, and spindle or stellate cells. Microscopically, in the pale mucus-like background, spindle or stellate cells were scattered or arranged in short bundles, and the nuclei of spindle cells were full and ovoid, and the interstitium was infiltrated with inflammatory cells.	Diagnostic differentiation relies on positive immunohistochemical reactions for calponin, smooth muscle actin (SMA), and desmin in myofibroblast (21).
RH	RH are rare and clinically heterogeneous histiocytic disorders of dermatological interest. RH may involve a single system or multiple systems. Single-system unifocal RH is the most common type of RH in clinical practice, occurring mainly in the skin, and has not been reported in the lungs (22).	Large epithelioid mononuclear or binucleated cells with round to kidney-shaped nuclei are seen microscopically. Sometimes invaginations of peripheral lymphocytes and neutrophils are seen.	Tumor cells is positive for CD68, CD 163, factor XIIIa and CD 14, and usually the Ki-67 proliferation index is low. RH and APH are hardly distinguishable by morphology and immunophenotype, and the final diagnosis needs to be made on the basis of detailed patient data.
Histiocytic Sarcoma	It usually involve lymph node sites, including the gastrointestinal tract, superficial and deep soft tissues, lungs, and nasal cavity. Lymph nodes, skin, and brain have also been reported.	Histiocytic sarcoma is an extremely rare malignant tumor characterized morphologically and immunophenotypically by histiocytes. Morphology, abundant eosinophilic to vacuolated or foamy cytoplasm, ovoid to irregularly shaped nuclei, and distinct nucleoli. Mitotic activity and tumor necrosis were evident, and a mixed background inflammatory infiltrate was frequently evident.	Molecular testing reveals clonal IgH or TCR gene rearrangements. The disease overlaps with the APH immunophenotype and needs to be differentiated by histologic morphology and molecular testing (23).

BHD must be differentiated from other diffuse cystic lung diseases. Lymphangioleiomyomatosis (LAM), common in women of childbearing age with TSC1/2 mutations, shows multiple nodules of immature smooth muscle and perivascular epithelial cells. It is positive for HMB-45 and SMA, and partially reactive for estrogen receptor (ER) and progesterone receptor (PR).

Lymphocytic Interstitial Pneumonia (LIP) is marked by polyclonal lymphocyte proliferation and presents as diffuse cystic lung lesions. Differentiating LIP from BHD is usually straightforward, as BHD lacks LIP’s specific features.

### Prognosis

The rarity and variability of APH complicate the standardization of treatment protocols. Current options include surgical excision, chemotherapy, ALK inhibitors, and supportive measures like blood transfusions. Currently, patients with APH involving only a single system rarely relapse after surgical resection. ALK inhibitors are more commonly used in patients with multi-organ involvement who cannot undergo surgical resection. Prognosis varies widely, with some patients experiencing spontaneous regression under supportive care, while others face relapse or mortality after initial treatment. Despite promising results from various ALK inhibitors, the limited number of cases and lack of definitive guidelines mean that ongoing evaluation and data collection are essential for optimizing patient prognosis and treatment strategies. However, since the patient did not receive drug therapy, the efficacy associated with ALK inhibitors needs to be further explored.

Management of BHD-associated cystic lung disease lacks specific therapies, and the effectiveness of mTOR inhibitors in preventing cyst formation is unclear. Thus, treatment focuses on preventing and managing pneumothorax.

In summary, the co-occurrence of histiocytic tumors and BHD is rare and requires careful evaluation of epithelioid, foamy, and spindle cell patterns. Accurate diagnosis depends on combining morphological assessment, immunohistochemistry, and molecular testing. It is crucial to consider family history and related cutaneous and renal pathology, especially in patients with pulmonary bullae without distinct pathological features, and to screen for FLCN gene mutations. This case highlights a broader range of onset ages for APH and represents the first known instance of these two rare conditions occurring together. Pathologists should consider the potential link between nodular and cystic lesions in APH and use second-generation sequencing to explore possible correlations. Enhanced awareness and expertise in distinguishing histiocytic tumors and BHD are essential for preventing misdiagnosis and ensuring effective patient care.

## Data Availability

The raw data supporting the conclusions of this article will be made available by the authors, without undue reservation.

## References

[1] Emile JF Abla O Fraitag S Horne A Haroche J Donadieu J. Revised classification of histiocytoses and neoplasms of the macrophage-dendritic cell lineages. Blood. (2016) 127:2672–81. doi: 10.1182/blood-2016-01-690636 PMC5161007 26966089

[2] Choi JK Xiao W Chen X Loghavi S Elenitoba-Johnson KS Naresh KN. Fifth edition of the world health organization classification of tumors of the hematopoietic and lymphoid tissues: acute lymphoblastic leukemias, mixed-phenotype acute leukemias, myeloid/lymphoid neoplasms with eosinophilia, dendritic/histiocytic neoplasms, and genetic tumor syndromes. Mod Pathol. (2024) 37:100466. doi: 10.1016/j.modpat.2024.100466 38460674

[3] Kemps PG Picarsic J Durham BH Helias-Rodzewicz Z Hiemcke-Jiwa L van den Bos C. ALK-positive histiocytosis: a new clinicopathologic spectrum highlighting neurologic involvement and responses to ALK inhibition. Blood. (2022) 139:256–80. doi: 10.1182/blood.2021013338 PMC8759533 34727172

[4] Zou L Lu T Li M Wang A Zhang Z Pan B. Localised ALK-positive histiocytosis in lung with EML4::ALK fusion. Pathology. (2024) 56:604–06. doi: 10.1016/j.pathol.2023.09.014 38097451

[5] Guo Y Qu HB Ning G Jia FL Liu H Ma XM. Case report: ALK-positive histiocytosis with KIF5B-ALK fusion in cerebrum-disseminated lesions in a child. Front Oncol. (2022) 12:858939. doi: 10.3389/fonc.2022.858939 35359354 PMC8960947

[6] Xu J Huang X Wen Y Pan Z Lian H Zhao M. Systemic juvenile xanthogranuloma has a higher frequency of ALK translocations than BRAFV600E mutations. J Am Acad Dermatol. (2023) 88:656–59. doi: 10.1016/j.jaad.2020.08.053 32822792

[7] Beck EM Bauman TM Rosman IS. A tale of two clones: Caldesmon staining in the differentiation of cutaneous spindle cell neoplasms. J Cutan Pathol. (2018) 45:581–87. doi: 10.1111/cup.13259 29687929

[8] Akimoto E Tokunaga M Sato R Yoshida A Naito Y Yamashita R. Gastric mesenchymal tumor with smooth muscle differentiation and echinoderm microtubule-associated protein-like 4-anaplastic lymphoma kinase (EML4-ALK) fusion. Pathol Int. (2021) 71:707–11. doi: 10.1111/pin.13154 34432920

[9] Khatri A Agrawal A Sikachi RR Mehta D Sahni S Meena N. Inflammatory myofibroblastic tumor of the lung. Adv Respir Med. (2018) 86:27–35. doi: 10.5603/ARM.2018.0007 29490419

[10] Bai Y Sun W Niu D Yang X Diao X Yu Y. Localized ALK-positive histiocytosis in a Chinese woman: report of a case in the lung with a novel EML4-ALK rearrangement. Virchows Arch. (2021) 479:1079–83. doi: 10.1007/s00428-021-03092-8 33825946

[11] Liu W Liu HJ Wang WY Tang Y Zhao S Zhang WY. Multisystem ALK-positive histiocytosis: a multi-case study and literature review. Orphanet J Rare Dis. (2023) 18:53. doi: 10.1186/s13023-023-02649-x 36915094 PMC10010018

[12] Feng X Tao J He N Wang J He L Zhang N. ALK-positive histiocytosis in 12 Asian children. Hum Pathol. (2024) 152:105637. doi: 10.1016/j.humpath.2024.105637 39117024

[13] Kolik AK Bakkaloglu DV Yilmaz I Cakir MS Yegen G Kara M. Incidentally detected ALK-positive histiocytosis with EML4::ALK fusion in a solitary pulmonary nodule following COVID-19 infection: A rare case report. Int J Surg Pathol. (2024) 104932276. doi: 10.1177/10668969241271372 39275853

[14] Yuan CT Chen JS Huang YL Zhang MS Hsieh MS. ALK-positive histiocytosis presenting as a solitary pulmonary nodule. Br J Haematol. (2022) 199:7. doi: 10.1111/bjh.18371 35867481

[15] Daccord C Good JM Morren MA Bonny O Hohl D Lazor R. Birt-hogg-dube syndrome. Eur Respir Rev. (2020) 29:200042. doi: 10.1183/16000617.0042-2020 32943413 PMC9489184

[16] Clave P Cabib C Ortega O. Cortical metaplasticity as a novel candidate mechanism for boosting brain swallow performance in neurogenic dysphagia. J Physiol. (2020) 598:5003–04. doi: 10.1113/JP280663 32949413

[17] Janssen D Harms D. Juvenile xanthogranuloma in childhood and adolescence: a clinicopathologic study of 129 patients from the kiel pediatric tumor registry. Am J Surg Pathol. (2005) 29:21–8. doi: 10.1097/01.pas.0000147395.01229.06 15613853

[18] Shi SS Sun YT Guo L. Rosai-Dorfman disease of lung: a case report and review of the literatures. Chin Med J (Engl). (2009) 122:873–74. 19493405

[19] Ravindran A Rech KL. How I diagnose rosai-dorfman disease. Am J Clin Pathol. (2023) 160:1–10. doi: 10.1093/ajcp/aqad047 37167084

[20] Haroche J Cohen-Aubart F Amoura Z. Erdheim-chester disease. Blood. (2020) 135:1311–18. doi: 10.1182/blood.2019002766 32107533

[21] McDermott M. Inflammatory myofibroblastic tumour. Semin Diagn Pathol. (2016) S0740-2570(16)30066-1. doi: 10.1053/j.semdp.2016.08.007 28029513

[22] Bonometti A Berti E. Reticulohistiocytoses: a revision of the full spectrum. J Eur Acad Dermatol Venereol. (2020) 34:1684–94. doi: 10.1111/jdv.16214 31955466

[23] Hung YP Qian X. Histiocytic sarcoma. Arch Pathol Lab Med. (2020) 144:650–54. doi: 10.5858/arpa.2018-0349-RS 31070934

