# Supplementary material for: A case of pulmonary ALK-positive histiocytosis combined with Birt-Hogg-Dubé syndrome carrying an EML4::ALK gene fusion: a case report and literature review
Source: Front Immunol. 2025 Jan 10;15:1501217. doi: 10.3389/fimmu.2024.1501217 (PMC11757237; doi:10.3389/fimmu.2024.1501217)
Supplement: Supplementary file 1 [file DataSheet1.docx]

### 2013 CARE Checklist

1. **Title-The diagnosis or intervention of primary focus followed by the words “case report”.**

**A case of pulmonary ALK-positive histiocytosis combined with Birt-Hogg-Dubé syndrome carrying an EML4-ALK gene fusion**

1. **Key Words-2 to 5 key words that identify diagnoses or interventions in this case report (including "case report").**

ALK-positive histiocytosis, Birt-Hogg-Dubé syndrome, EML4-ALK gene fusion, FLCN gene, case report

1. **Abstract-(structured or unstructured)**

**3a. Introduction – What is unique about this case and what does it add to the scientific literature?**

This article is the first to report the combined existence of APH and BHD, and pathologists need to pay close attention to them.

**3b. The patient’s main concerns and important clinical findings.**

APH and BHD are very rare, and it is easy to misdiagnose or miss the diagnosis altogether if one is not familiar with the associated histology and immunohistochemistry.

**3c. The primary diagnoses, interventions, and outcomes.**

APH and BHD. The patient underwent wedge resection of the lower lobe of the right lung. During follow-up, no further treatment was given, and there was no evidence of recurrence or metastasis at the 8-month follow-up.

**3d. Conclusion-What are one or more “take-away” lessons from this case report?**

It is essential for pathologists to recognize the presence of these two diseases and understand the associated histomorphologic, immunohistochemical, and cytogenetic features to enable an accurate diagnosis and differential diagnosis.

1. **Introduction-Briefly summarizes why this case is unique and may include medical literature references.**

This article is the first to report the combined existence of APH and BHD, and pathologists need to pay close attention to them.

1. **Patient Information**

**5a. De-identified patient specific information.**

A 61-year-old woman

**5b. Primary concerns and symptoms of the patient.**

A 61-year-old woman presented in August 2023 with fever, dry cough, chest tightness, shortness of breath, and pain. On September 22, 2023, a chest CT scan revealed multiple alveoli in both lungs, nodules in the right lower lobe.

**5c. Medical, family, and psychosocial history including relevant genetic information.**

inapplicability

**5d. Relevant past interventions and their outcomes.**

Inapplicability

1. **Clinical Findings-Describe significant physical examination (PE) and important clinical findings.**

There are no typical significant clinical findings in this patient.

1. **Timeline-Historical and current information from this episode of care organized as a timeline (figure or table).**

| Time | August 2023 | September 22, 2023 | October 19, 2023 |
| --- | --- | --- | --- |
| current information | fever, dry cough, chest tightness, shortness of breath, and pain | a chest CT scan revealed multiple alveoli in both lungs, nodules in the right lower lobe | She underwent a right lower lobe wedge resection |

1. **Diagnostic Assessment**

**8a. Diagnostic methods (PE, laboratory testing, imaging, surveys).**

laboratory testing

**8b. Diagnostic challenges.**

In this case, the patient tested positive for Caldesmon and negative for SMA and Desmin, possibly due to Caldesmon's higher sensitivity. CD68 and CD163 expression varied across tumor sections, with some showing strong positivity and others resembling atypical ALK-rearranged histiocyte-rich tumors.

**8c. Diagnosis (including other diagnoses considered).**

**ALK-positive histiocytosis combined with Birt-Hogg-Dubé syndrome carrying an EML4-ALK gene fusion**

**8d. Prognostic characteristics when applicable.**

Current options include surgical excision, chemotherapy, ALK inhibitors, and supportive measures like blood transfusions. Prognosis varies widely, with some patients experiencing spontaneous regression under supportive care, while others face relapse or mortality after initial treatment.

1. **Therapeutic Intervention**

**9a. Types of therapeutic intervention (pharmacologic, surgical, preventive).**

surgical

**9b. Administration of therapeutic intervention (dosage, strength, duration).**

This patient did not undergo treatment other than surgery.

**9c. Changes in therapeutic interventions with explanations.**

This patient did not undergo treatment other than surgery.

1. **Follow-up and Outcomes**

**10a. Clinician- and patient-assessed outcomes if available.**

During follow-up, no further treatment was given, and there was no evidence of recurrence or metastasis at the 8-month follow-up.

**10b. Important follow-up diagnostic and other test results.**

There was no evidence of recurrence or metastasis at the 8-month follow-up.

**10c. Intervention adherence and tolerability. (How was this assessed?)**

inapplicability

**10d. Adverse and unanticipated events.**

inapplicability

1. **Discussion**

**11a. Strengths and limitations in your approach to this case.**

Accurate diagnosis depends on combining morphological assessment, immunohistochemistry, and molecular testing. It is crucial to consider family history and related cutaneous and renal pathology, especially in patients with pulmonary alveoli without distinct pathological features, and to screen for FLCN gene mutations.

**11b. Discussion of the relevant medical literature.**

1. Chan JK, Lamant L, Algar E, Delsol G, Tsang WY, Lee KC, et al. ALK+ histiocytosis: a novel type of systemic histiocytic proliferative disorder of early infancy. *Blood* (2008) **112**: 2965-68. doi:10.1182/blood-2008-03-147017

2. Kemps PG, Picarsic J, Durham BH, Helias-Rodzewicz Z, Hiemcke-Jiwa L, van den Bos C, et al. ALK-positive histiocytosis: a new clinicopathologic spectrum highlighting neurologic involvement and responses to ALK inhibition. *Blood* (2022) **139**: 256-80. doi:10.1182/blood.2021013338

3. Zou L, Lu T, Li M, Wang A, Zhang Z, Pan B, et al. Localised ALK-positive histiocytosis in lung with EML4::ALK fusion. *Pathology* (2024) **56**: 604-06. doi:10.1016/j.pathol.2023.09.014

4. Beck EM, Bauman TM, Rosman IS. A tale of two clones: Caldesmon staining in the differentiation of cutaneous spindle cell neoplasms. *J Cutan Pathol* (2018) **45**: 581-87. doi:10.1111/cup.13259

5. Akimoto E, Tokunaga M, Sato R, Yoshida A, Naito Y, Yamashita R, et al. Gastric mesenchymal tumor with smooth muscle differentiation and echinoderm microtubule-associated protein-like 4-anaplastic lymphoma kinase (EML4-ALK) fusion. *Pathol Int* (2021) **71**: 707-11. doi:10.1111/pin.13154

6. Clave P, Cabib C, Ortega O. Cortical metaplasticity as a novel candidate mechanism for boosting brain swallow performance in neurogenic dysphagia. *J Physiol* (2020) **598**: 5003-04. doi:10.1113/JP280663

7. Janssen D, Harms D. Juvenile xanthogranuloma in childhood and adolescence: a clinicopathologic study of 129 patients from the kiel pediatric tumor registry. *Am J Surg Pathol* (2005) **29**: 21-28. doi:10.1097/01.pas.0000147395.01229.06

8. Ravindran A, Rech KL. How I Diagnose Rosai-Dorfman Disease. *Am J Clin Pathol* (2023) **160**: 1-10. doi:10.1093/ajcp/aqad047

9. Haroche J, Cohen-Aubart F, Amoura Z. Erdheim-Chester disease. *Blood* (2020) **135**: 1311-18. doi:10.1182/blood.2019002766

10. Allen CE, Merad M, McClain KL. Langerhans-Cell Histiocytosis. *N Engl J Med* (2018) **379**: 856-68. doi:10.1056/NEJMra1607548

11. McDermott M. Inflammatory myofibroblastic tumour. *Semin Diagn Pathol* (2016). doi:10.1053/j.semdp.2016.08.007

**11c. The rationale for your conclusions.**

morphological assessment, immunohistochemistry, and molecular testing

**11d. The primary “take-away” lessons from this case report (without references) in a one paragraph conclusion.**

This case highlights a broader range of onset ages for APH and represents the first known instance of these two rare conditions occurring together.

1. **Patient Perspective-The patient should share their perspective on the treatment(s) they received.**

inapplicability

1. **Informed Consent-The patient should give informed consent. (Provide if requested.)**

yes
